# Scalable-produced 3D elastic thermoelectric network for body heat harvesting

**DOI:** 10.1038/s41467-023-38852-4

**Published:** 2023-05-27

**Authors:** Yijie Liu, Xiaodong Wang, Shuaihang Hou, Zuoxu Wu, Jian Wang, Jun Mao, Qian Zhang, Zhiguo Liu, Feng Cao

**Affiliations:** 1 School of Physics, Harbin Institute of Technology, Harbin, 150001 PR China; 2 School of Science, and Ministry of Industry and Information Technology Key Lab of Micro-Nano Optoelectronic Information System, Harbin Institute of Technology, Shenzhen, 518055 PR China; 3 School of Materials Science and Engineering, Institute of Materials Genome & Big Data, and Flexible Printed Electronics Technology Center, Harbin Institute of Technology, Shenzhen, 518055 PR China; 4 State Key Laboratory of Advanced Welding and Joining, Harbin Institute of Technology, Harbin, 150001 PR China

**Keywords:** Thermoelectric devices and materials, Porous materials, Thermoelectrics, Devices for energy harvesting

## Abstract

Flexible thermoelectric generators can power wearable electronics by harvesting body heat. However, existing thermoelectric materials rarely realize high flexibility and output properties simultaneously. Here we present a facile, cost-effective, and scalable two-step impregnation method for fabricating a three-dimensional thermoelectric network with excellent elasticity and superior thermoelectric performance. The reticular construction endows this material with ultra-light weight (0.28 g cm^−3^), ultra-low thermal conductivity (0.04 W m^−1^ K^−1^), moderate softness (0.03 MPa), and high elongation (>100%). The obtained network-based flexible thermoelectric generator achieves a pretty high output power of 4 μW cm^−2^, even comparable to state-of-the-art bulk-based flexible thermoelectric generators.

## Abstract (web summary)

Flexible thermoelectric generators can use body heat to power electronic wearables but are often limited by a trade-off between flexibility and output performance. Here, authors demonstrate a scalable, lightweight, elastic, and high-performing network-based Ag2Se thermoelectric generator.

## Introduction

With the rapid development of “Internet of Things” technology, the popularity of flexible electronics has risen enormously. As a result, the global flexible electronics market was valued at USD ~28 billion in 2020 and that is expected to reach USD ~ 56 billion by 2026^1^. However, the convenience and endurance of the most common flexible electronics are hindered by their power sources which need to be able to operate continuously^2^. Various energy-harvesting technologies, such as triboelectric nanogenerators^3,4^, piezoelectric nanogenerators^5^, flexible solar cells^6,7^, and flexible thermoelectric generators (FTEG)^2,8^, were thus developed. Features that directly convert body heat into electricity make the FTEG highly appealing, as power is sustainably generated without the need for chemical energy, sunlight, or mechanical motion^9–11^.

The common bulk thermoelectric materials, whether inorganic materials (e.g., bismuth antimony telluride alloys^12^ and silver selenide^13^, etc.) or conducting polymers [e.g., PEDOT:PSS (poly (3,4-ethylenedioxythiophene):poly (styrene sulfonate))^14–16^ and PANI (polyaniline)^15,17–19^], are intrinsically inflexible^20^. Some strategies were thus proposed to meet the flexibility of FTEG. One strategy is processing the thermoelectric material into thin film or sheet^21–29^. Although bendable^26,27,30^ or even elastic^31^, the thermoelectric thin film or sheet usually harvests thermal energy in the in-plane direction rather than in the more desirable film-thickness direction. Three-dimension (3D) FTEG, by contrast, is more suitable for harvesting body heat during wear. Combining flexible matrix with rigid thermoelectric legs is a device-level strategy to obtain 3D FTEGs^32–37^. This kind of FTEG has the highest thermoelectric performance, but the heavy and incompatible rigid thermoelectric legs significantly reduce their wearing comfort and reliability^9,38^. To better match the elastic skin, improve comfortability, and recover the deformation induced in operation, developing a thermoelectric material with elasticity is necessary for an FTEG. Mixing insulating elastomers with conducting polymers or carbon nanotubes can result in 3D stretchable thermoelectric materials or thermoelectric elastomers^18,20,39–41^. However, these composites demonstrate inferior thermoelectric performance (*zT* < 0.03) in comparison to the inorganic thermoelectric materials due to their intrinsically low Seebeck coefficient^20^. Consequently, achieving a material simultaneously possesses elasticity and high thermoelectric performance is still challenging.

Here, we report a facile, cost-effective, and scalable two-step impregnation method for synthesizing a 3D Ag_2_Se network. Benefiting from the deformable and low-thermal-conductive network structure, the proposed thermoelectric network can not only exhibit sufficient elasticity (elongation > 100%), but also achieve a comparable output power to traditional bulk-based FTEGs with the flexible matrix. Unlike the conventional preparation process of thermoelectric material or device, our fabrication technique depends hardly on complex equipment, which makes the Ag_2_Se network can be large-scale produced at low cost and processed into special shapes to match the various heater surfaces. Furthermore, the soft texture (Young’s modulus ~0.03 MPa) and the ultralight weight (density ~0.28 g cm^−3^) of Ag_2_Se network also ensure its enormous potential in wearable applications. A thermoelectric network-filled jacket is fabricated to verify the actual effect of body heat harvesting. It can generate milliwatt-level power in daily wear, covering the consumption of most integrated circuits and sensors in wearables^42,43^. The high output performance, scalable technique, and significant advantages in wearability enable the promising applications of Ag_2_Se network.

## Results and discussion

### Three-dimensional elastic Ag_2_Se network

**Fig. 1:**
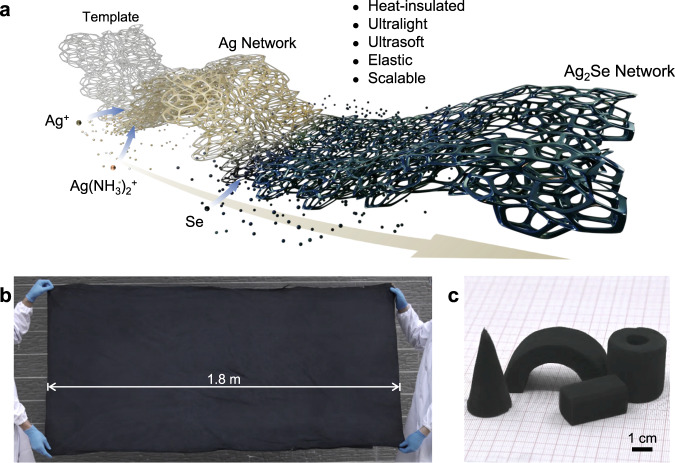
Fabrication process and photos of Ag_2_Se network. **a** Schematic of the two-step impregnation process, showing the in-situ synthesis of Ag_2_Se network by silvering and then selenizing. **b** A large Ag_2_Se network with a size of 1.8 × 0.9 m^2^. **c** Ag_2_Se networks are processed to special shapes.

Our two-step impregnation method for making the Ag_2_Se network (Fig. 1a) is inspired by two classical chemical reactions, “Tollens’ reaction”^44^ and “Transition metal selenization”^45^. In the first step, SnCl_2_-pretreated melamine template is immersed into Tollens’ reagent containing Ag^+^ and Ag(NH_3_)_2_^+^. The Ag^+^ in the solution is reduced by the Sn^2+^ on the template to form Ag nucleus^46^, and the coexisting Ag(NH_3_)_2_^+^ is subsequently reduced by the reductant comprising glucose monohydrate and potassium sodium tartrate tetrahydrate to form a silver network. In the second step, the obtained silver network is impregnated into the selenium solution^47^ for selenizing to in-situ synthesize the Ag_2_Se network^48^. The porosity of the Ag_2_Se network measured with the Archimedes method can be controlled from 98% to 95% with a pristine porosity of 99% through the adjustment of reaction proportion and reaction times (Fig. S1). The detailed optimization is provided in Experimental Section. Because all the procedures are handled at ambient conditions, a nearly 2-square-meter sample is effortlessly produced (Figs. 1b and S3). Furthermore, unlike brittle thermoelectric bulks, the obtained three-dimensional network can be easily processed into special shapes (e.g., arch, cone, cuboid, and tube, etc.) to match well with the complex heater surfaces (Fig. 1c).

**Fig. 2:**
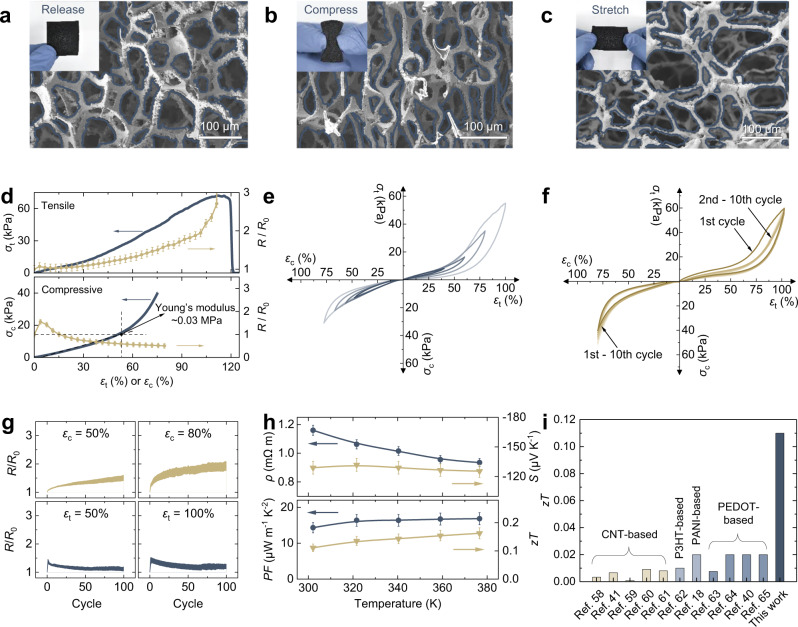
Mechanical, and thermoelectric properties of Ag_2_Se network. **a**–**c** Photos (inset) and cross-section micrographs of the Ag_2_Se network in different states, showing the deformation of network structure under stress. **d** Tensile stress (*σ*_t_)–strain (*ε*_t_) curve and compressive stress (*σ*_c_)–strain (*ε*_c_) curve with corresponding resistance change (*R*/*R*_0_). **e** Cyclic *σ*_c_–*ε*_c_ curves with strain varying from 50% to 80% (bottom left), and *σ*_t_–*ε*_t_ curves with strain from 40% to 100% (top right). **f** Cyclic strain-stress curves at a maximum *ε*_c_ of 80% (bottom left) and a maximum *ε*_t_ of 100% (top right). **g** *R*/*R*_0_ upon cyclic compressing (top) and stretching (bottom) under different strain rates. **h** Thermoelectric performance of Ag_2_Se network including electrical resistivity (*ρ*), Seebeck coefficient (*S*), power factor (*PF*), and *zT* value. **i** Comparison of the present *zT* value with reported 3D flexible thermoelectric materials. The inset shows the larger version of the *zT* values of CNT-based materials. Error bars represent the standard deviation. Source data are provided as a Source Data file.

With the help of the micro-scale structure deformation, the Ag_2_Se network demonstrates macro-scale stretching and compressing (Fig. 2a–c), which is similar to the metal-mesh electrodes characterized in the previous works^49^. A tensile strain (*ε*_t_) of >100% and a compressive strain (*ε*_c_) of >80% were obtained (Fig. 2d). The Young’s modulus of the Ag_2_Se network extracted from the compressive stress-strain curve is ~0.03 MPa, which is considered as an ideal and tissue-like modulus that enables the intimate fitting of devices with the uneven skin, reducing the effects of mechanical loads^50–53^. The room-temperature Seebeck coefficient and resistivity of the Ag_2_Se network under compressive and tensile strains were measured on a ZEM-3. The results indicate that there is little change in the Seebeck coefficient with the strain, and the change in power factor mainly results from the change in resistivity, which can be ascribed to the variation in the geometric size of the Ag_2_Se network. (Fig. S4). We also carried out cyclic mechanical and electromechanical tests to show the durability of the Ag_2_Se network (Fig. 2e–g). Even though a hysteresis in the stress-strain curve is observed because of viscoelasticity, the sample can recover to its initial state after the successive cycles at different strains (Fig. 2e). A slight mechanical degradation occurs in the initial cycle of the tensile-strain test (Fig. 2f), but it does not deteriorate in the subsequent cycles. Correspondingly, the relative resistance of the Ag_2_Se network almost remained stable under cyclic tests with different strain rates (Fig. 2g). The changes in resistance are similar to the typical trend of stretchable conductive materials^31,54^. The possible reasons for the decline in resistance over cycles are the alignment of fibrils and the viscoelasticity of the template^31,55^. The Ag_2_Se network, as an elastic material, also performs well in bending. It can survive 200 cycles bending with a 5 mm radius without significant deterioration (Fig. S5). Additional characterizations of the Ag_2_Se network including morphology, energy dispersive spectrum, element mapping, XRD pattern, and optical bandgap are provided in Fig. S6–S10 and Table S1. The obtained Ag_2_Se network is a single phase without any impurity, and the bandgap is about 0.23 eV.

Together with good mechanical properties, the Ag_2_Se network also possesses superior thermoelectric performance (Fig. 2h). It displays a similar Seebeck coefficient (~−130 μV K^−1^) and much lower thermal conductivity (~0.04 W m^−1^ K^−1^, Fig. S11) compared with that of Ag_2_Se bulk materials at room temperature^56,57^. A high room-temperature *zT* ~ 0.11 is achieved, which is one or two orders of magnitude higher than the previously reported three-dimensional flexible thermoelectric materials based on polymer composites^18,40,41,58–65^ (Fig. 2i), indicating that structuring inorganic material is also another viable pathway to develop three-dimensional flexible thermoelectric materials.

### Ag_2_Se network-based device

**Fig. 3:**
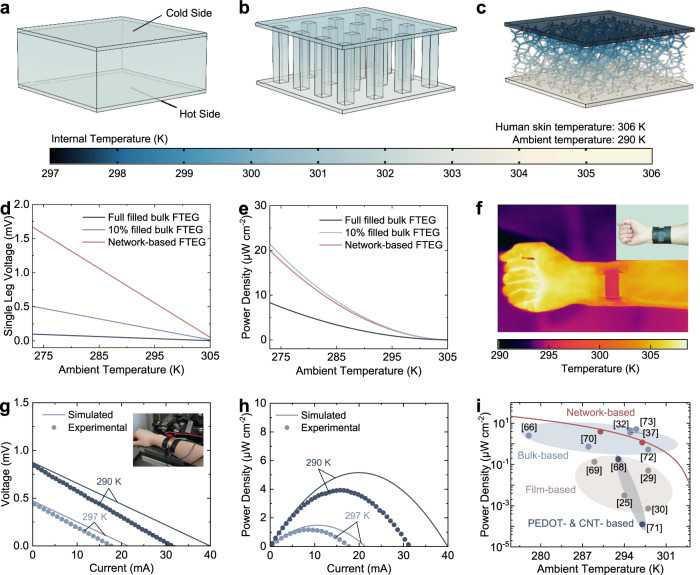
Output performance of Ag_2_Se network-based FTEG. The simulated internal temperature distribution of the full filled (**a**), 10% filled (**b**) bulk FTEGs and the network-based FTEG (**c**) at an ambient temperature of 290 K. Further simulation details are provided in the supplementary materials. **d**, **e**, The simulated single leg voltage and power density in an ambient temperature range from 273 K to 305 K. **f** The infrared image and optical photograph (inset) of a network-based FTEG dressed on a human wrist. The top electrode was removed to avoid the effect caused by its low emissivity. **g**, **h** The measured voltage and power density at an ambient temperature of 290 K and 297 K. Inset shows a device under test. **i** Summary of the FTEG’s power generation at different ambient temperatures. All the measurements were performed on human skin without heat sinks. Red line and red circles respectively represent the simulated and measured values of the Ag_2_Se network-based FTEG. Source data are provided as a Source Data file.

The ultralow thermal conductivity of the Ag_2_Se network is highly desirable for the FTEG. First, it endows the FTEG with a superior ability to establish temperature difference, unlike the bulk-based FTEG, which has to enlarge the temperature difference by increasing the leg height, decreasing the fill factor (the percentage of total FTEG area occupied by the thermoelectric legs) or imposing a heat sink. This advantage is native to the network-based FTEG without additional weight, volume, or power. By finite element simulation, we compared the temperature distribution between the network-based FTEG, the full-filled bulk-based FTEG and the 10% filled FTEG at the same thickness of 3 mm (Fig. 3a–c). The completely filled and 10% filled bulk-based FTEGs are made up of Bi_2_Te_3_-based thermoelectric legs and PDMS (Polydimethylsiloxane) matrix, which are common in the previous works^66,67^. The network-based FTEG shows a large temperature difference of ~6.5 K, one order higher than the full-filled bulk-based FTEG and three times higher than the 10% filled FTEG. Second, the Ag_2_Se network-based FTEG can maintain normal body surface temperature during operation, which is essential but often unconsidered. To maximize the output power, it is better to use FTEGs in a cold environment. Suffering from the high thermal conduction or the heat sink cooling, the temperature drop in body surface caused by the low hot-side temperature is a significant complication for bulk-based FTEG, which can be avoided in the network-based FTEG as its interior low thermal conduction can prevent heat loss. According to the simulated temperature field (Fig. 3a–c), the network-based FTEG’s hot side temperature (~304 K) is closer to skin temperature (306 K) than the bulk-based FTEGs. We also compared the output performance by simulation at different ambient temperatures from 273 K to 305 K (Fig. 3d, e). A great increase in the open-circuit voltage of the network-based FTEG appears in comparison to the bulk-based FTEGs due to the larger temperature difference (Fig. 3d). Consequently, the power density (output power per unit area) of the network-based FTEG is similar to that of the 10% filled FTEG (Fig. 3e). The infrared image of the network-based FTEG wore on the arm experimentally demonstrates the obvious low temperature on the Ag_2_Se network’s surface (Fig. 3f). Although the tested voltage and power density of a network-based FTEG attached on the arm are slightly lower than the simulated results (Fig. 3g, h), which is because of the contact resistance, the power density of network-based FTEG is several order higher than that of PEDOT- or CNT-based FTEGs and is even on par with high-performance bulk-based FTEGs^25,29,32,37,66,68–73^ (Fig. 3i). Additionally, the device demonstrates excellent reliability and can operate continuously for 50 h without degradation in output performance (Fig. S12).

**Fig. 4:**
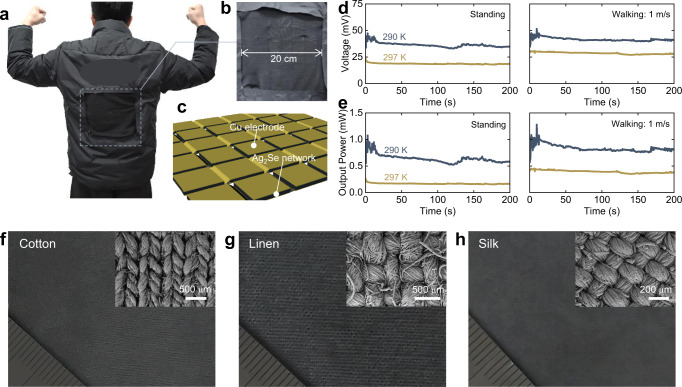
Thermoelectric jacket and fabrics. **a** A thermoelectric jacket filled with the Ag_2_Se network-based FTEG. **b** Detailed photograph of the thermoelectric-device area. **c** Schematic of the thermoelectric modules in series. **d,** **e** The thermoelectric jacket’s open-circuit voltage and output power under different thermal conditions. **f**–**h** The optical photos and morphologies (inset) of various thermoelectric fabrics. Further micrographs and thermoelectric performances are shown in Figs. S13 and S14. Ruler scale unit: mm. Source data are provided as a Source Data file.

The excellent output performance of network-based FTEG is complemented by portability and scalability, which encourages us to take one step further toward FTEG’s practical use. We replaced a portion of the filler (20 × 20 cm^2^) in a commercial jacket with the network-based FTEG with 40 legs in series to verify the actual effect (Fig. 4a–c and Fig. S15). Promisingly, most of the device’s performance can be preserved even under such a non-ideal condition including the presence of hand-arranged module gaps, and the untight touching between the module’s hot side and skin surface (Fig. 4, d, e). When the wearer was sitting stationary in an indoor environment at 290 K, the maximum power of the thermoelectric jacket was ~0.6 mW. The output power increased to ~1 mW when the wearer was walking at a speed of 1 m s^−1^. In addition, although the thermal bypass in the current configuration of the FTEG module based on one-type TE leg has a negligible effect on the temperature difference (Fig. S16), the increased internal resistance is inevitable. To further enhance the output power, a thick electrode would be utilized in this module, resulting in the larger thermal bypass. We assembled the device with two p-type Bi_2_Te_3_-based legs and two n-type Ag_2_Se-based networks (Device size: 4.1 × 2.6 cm^2^) to estimate the possible performance of the device, and the tested results are shown in Fig. S17. As expected, the TEG with an n-p design exhibits higher performance. A voltage of ~15 mV and a power density of ~1.7 μW cm^−2^ can be reached, even if its geometric is not particularly optimized, exceeding the performance of the presented single-leg device (with a voltage of ~0.44 mV and a power density of ~1.2 μW cm^−2^) at the same ambient temperature of 297 K. However, the weight and portability of the TEG with rigid p-type Bi_2_Te_3_ would have deteriorated in comparison with the network-based device. Consequently, developing matched flexible p-type materials is really necessary.

The two-step impregnation method, shown here for the Ag_2_Se network, can also be applied to fabric substrates, including cotton, linen, and silk (Fig. 4f–h and Figs. S11, S18, S19). The technique thus allows ordinary cloth or clothing to be easily processed into personalized thermoelectric devices for the power supply or thermoregulation. The diverse possibilities enable the two-step impregnation technique, a universal strategy for making wearable thermoelectric products. Additionally, some compatible techniques or materials are deserved to be investigated. For instance, substituting liquid metal electrodes for copper electrodes to meet the Ag_2_Se network’s elasticity, and developing p-type thermoelectric materials using similar techniques to pair with n-type Ag_2_Se.

## Methods

### Preparation of the three-dimensional Ag_2_Se network and the network-based FTEG

#### Template preprocessing

A high-density melamine foam (Basotect; BASF) was first rinsed in NaOH solution (80 g/L) for 30 min, and then cleaned with deionized water. Tin (II) chloride dihydrate (SnCl_2_·2H_2_O, 98%; Innochem) was dissolved in deionized water (40 g/L) to make Sn^2+^ solution, followed by the addition of hydrochloric acid (AR; Xilong Scientific) until the solution is clear. The template was then immersed into Sn^2+^ solution for 2 h and transferred into deionized water for rinsing. Finally, the treated template dried naturally.

#### Silvering

Glucose monohydrate (C_6_H_12_O_6_·H_2_O, AR; Hushi), potassium sodium tartrate tetrahydrate (C_4_H_4_O_6_KNa·4H_2_O, AR, 99%; Aladdin), and PEG1000 (CP, Hushi) were dissolved in a diluted ethanol solution to make a reductant. The concentrations of C_6_H_12_O_6_·H_2_O, C_4_H_4_O_6_KNa·4H_2_O, PEG1000, and ethanol were 40 g/L, 14 g/L, 0.1 g/L, and 100 g/L, respectively. The Tollens’ reagent was prepared by dissolving silver nitrate (AgNO_3_, 98%; TBHX) and sodium hydroxide (NaOH, AR; Xilong Scientific) in an ammonium hydroxide solution (AR; Macklin). The aforementioned template was impregnated into the mixture of reductant and Tollens’ reagent, and squeezed gently to extrude the residual bubbles. After soaking for 5 h, the obtained silver network was washed and dried. It should be pointed out that the reaction rate and the number of reaction times will affect the silver loading and further influence the Ag_2_Se loading. Therefore, we adjusted the silver load by changing ammonia concentration and reaction times with a fixed AgNO_3_ concentration of 50 g/L and NaOH concentration of 25 g/L (Fig. S1).

#### Selenization

To prepare the selenium solution, sodium sulfide nonahydrate (Na_2_S·9H_2_O, 98%; Aladdin) and selenium powder (Se, 99.9%; Innochem) were dissolved into deionized water with concentrations of 60 g/L and 20 g/L, respectively. The above-mentioned silver network was then impregnated into the selenium solution for 10 h to synthesize the Ag_2_Se network. Considering the reaction efficiency, the subsequent samples were prepared based on an ammonia concentration of ~4.5 mol/L and two repetitions. The color changes in reaction process are shown in Fig. S2. The large-scale preparation was carried out in a polypropylene tank with a size of 2000 × 1000 × 100 mm^3^.

#### Fabrication of the network-based FTEG

The 10 μm-thick copper foils were used as electrodes, connected with the Ag_2_Se networks by silver paste (05002; SPI). A FTEG module including 40 thermoelectric legs of the Ag_2_Se networks was prepared in series, which was utilized to assemble a thermoelectric network-filled jacket by partially replacing the jacket’s filler.

### Characterization of the material and device

#### Material characterization

The porosity of the Ag_2_Se network was measured based on the Archimedes method by a density balance (BSA224S-CW; Sartorius). Morphology and energy dispersive spectrum of the Ag_2_Se network were observed by field emission scanning electron microscopy (Gemini Crossbeam 350; ZEISS). The crystal structure was determined by X-ray diffraction (SmartLab; Rigaku) using Cu Kα radiation in the 2*θ* range of 10–70° at 40 kV and 200 mA. The mechanical properties were tested by a stress–strain apparatus (HTS-LLY920C; Zhongye). The Seebeck coefficient (*S*) and electrical resistivity (*ρ*) of Ag_2_Se networks with or without strain were measured simultaneously on a commercial system (ZEM-3; Advance Riko). The room-temperature thermal conductivity (*κ*) was determined by the hot wire method on a thermal conductivity meter (TC3000E; XIATECH). The given results are the mean of three measurements taken from the same sample. Optical bandgap was obtained on a Fourier transform IR reflectance spectrometer (Nicolet iS50; Thermo Fisher Scientific) equipped with an integrating sphere coated with gold (Pike).

#### Simulation

The output performance for the bulk-based and network-based FTEGs was simulated using the thermoelectric module in COMSOL. All simulations were performed under natural convection conditions (Fig. S13). We also calculated the open-circuit voltage (*U*) and power density (*p*) of the network-based FTEG with different thicknesses. According to the simulated results (Fig. S14), a module thickness of 3 mm was adopted in the experiments. The material parameters used in the simulation were summarized in Table S2.

#### Network-based FTEG characterization

The output properties were measured in homemade equipment connected with two electricity meters (2400 and 2182; Keithley). The IR image was captured using a thermal IR imager (T620; FLIR). The ambient temperature and wind speed were recorded by a portable weather station (PWS; Qingyi).

### Ethics and inclusion statement

The authors declare that their study was approved by Harbin Institute of Technology (Protocol number: HIT-2022012), and they have obtained informed consent for publication from all participants.

### Reporting summary

Further information on research design is available in the Nature Portfolio Reporting Summary linked to this article.

## Supplementary information

Supplementary Information

Reporting Summary

## Data Availability

All data are available in the main text or the supplementary materials. Source data are provided with this paper.

## Source data

Source Data
