# Supplementary material for: Scalable-produced 3D elastic thermoelectric network for body heat harvesting
Source: Nat Commun. 2023 May 27;14:3058. doi: 10.1038/s41467-023-38852-4 (PMC10224941; doi:10.1038/s41467-023-38852-4)
Supplement: Supplementary file 1 — Supplementary Information [file 41467_2023_38852_MOESM1_ESM.pdf]

Supplementary Materials for

Scalable-Produced 3D Elastic Thermoelectric Network for Body Heat Harvesting

Yijie Liu[a, b, #], Xiaodong Wang[c, #], Shuaihang Hou[c], Zuoxu Wu[b], Jian Wang[b], Jun Mao[c, d], Qian Zhang[c, d, *], Zhiguo Liu[a, *], Feng Cao[b, *]

[a] *School of Physics, Harbin Institute of Technology, Harbin 150001, P. R. China* liuzhiguo@hit.edu.cn

[b] *School of Science, and Ministry of Industry and Information Technology Key Lab of Micro-Nano Optoelectronic Information System, Harbin Institute of Technology, Shenzhen 518055, P. R. China* caofeng@hit.edu.cn

[c] *School of Materials Science and Engineering, Institute of Materials Genome & Big Data, and Flexible Printed Electronics Technology Center, Harbin Institute of Technology, Shenzhen 518055, P. R. China* zhangqf@hit.edu.cn

[d] *State Key Laboratory of Advanced Welding and Joining, Harbin Institute of Technology, Harbin 150001, P. R. China*

[#] Equal contribution

Inventory of Supplementary Information:

- Supplementary Figures S1-S19 Page 2-20
- Supplementary Tables S1-S2 Page 21
- Supplementary References 1-4 Page 22

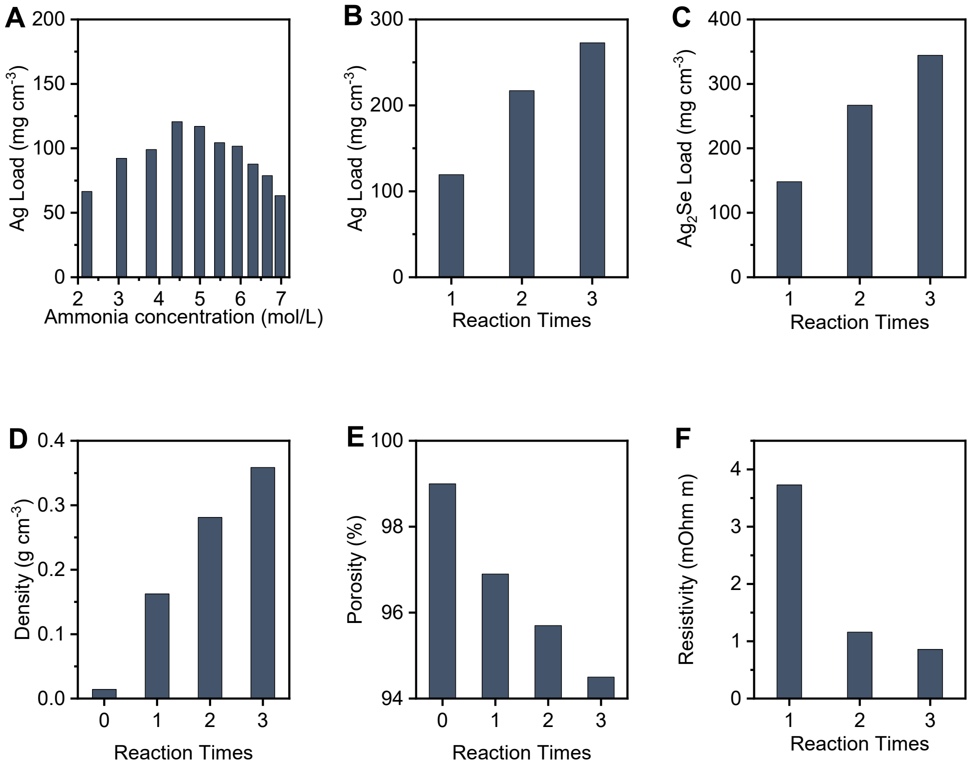

Fig. S1. **A**, The silver load with different ammonia concentration. The ammonia concentration with maximum loads was adopted for subsequent experiments. **B**, The silver load with different silvering times. **C**, The silver selenide load with different silvering times. **D**, The density of Ag_2Se network with different silvering times. The number of 0 represents the pristine melamine template before reaction. **E**, Porosity of the Ag_2Se networks were tested by the Archimedes method. **F**, Resistivity of the Ag_2Se network with different loads.

The principle of the Archimedes method is given as follows:

The weights of the dry sample in air (m_1), the sample fully filled with alcohol both in air (m_2) and in alcohol (m_3) were measured. Then the Ag_2Se network's volume V_{network} (excluding pores) can be calculated using

$$V_{\text{network}} = \frac{m_1 - m_3}{\rho_{\text{alcohol}}} \quad (1)$$

where ρ_{alcohol} is the density of alcohol. The pores' volume V_{pores} (excluding network) can be calculated using

$$V_{\text{pores}} = \frac{m_2 - m_1}{\rho_{\text{alcohol}}} \quad (2)$$

So, the porosity of the Ag_2Se network can be expressed as

$$\text{Porosity} = \frac{V_{\text{pores}}}{V_{\text{pores}} + V_{\text{network}}} = \frac{m_2 - m_1}{m_2 - m_3} \quad (3)$$

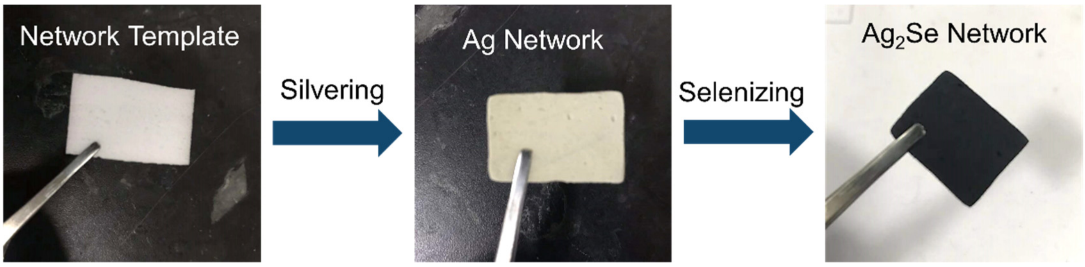

Fig. S2. The color changes in the reaction process.

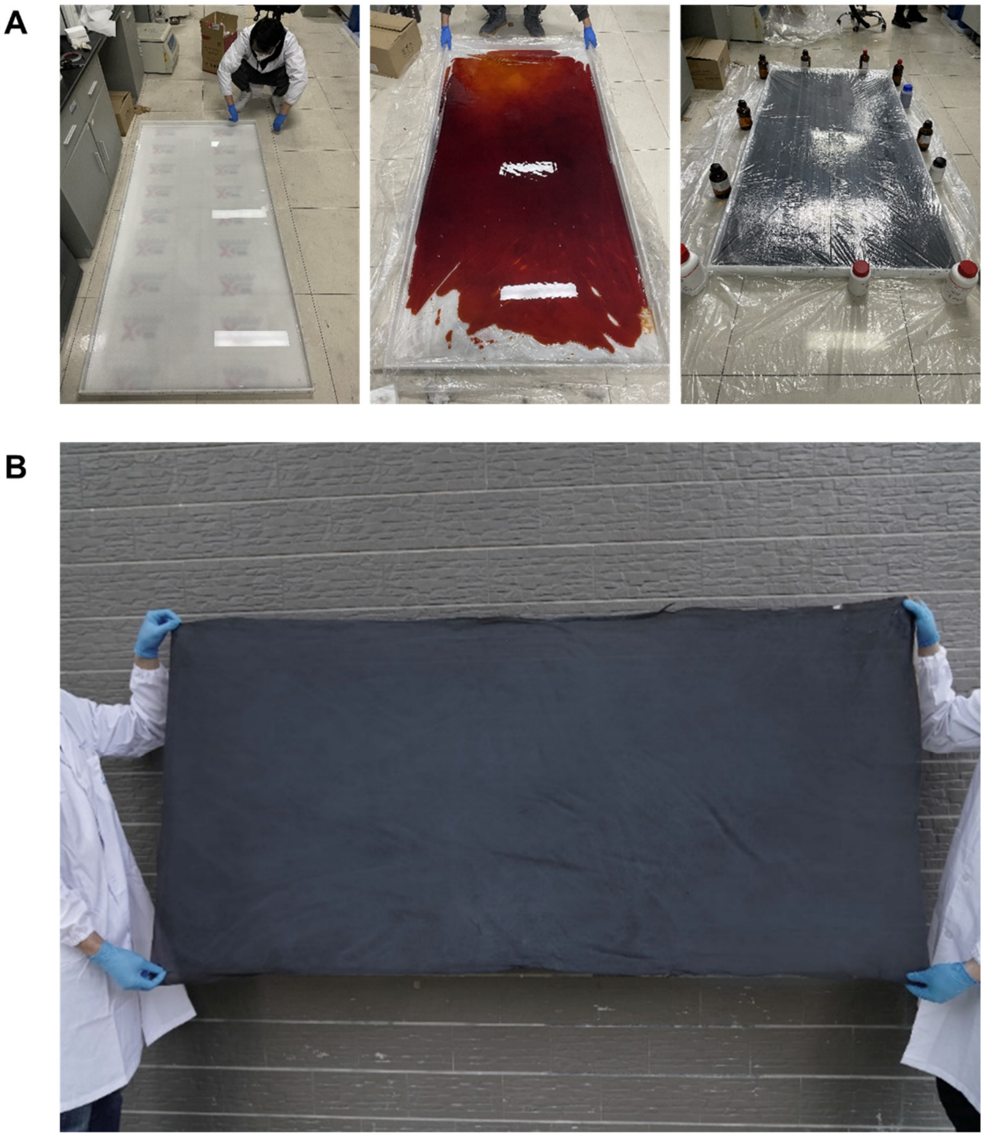

Fig. S3. **A**, The mass preparation process. **B**, The prepared large size Ag_2Se network.

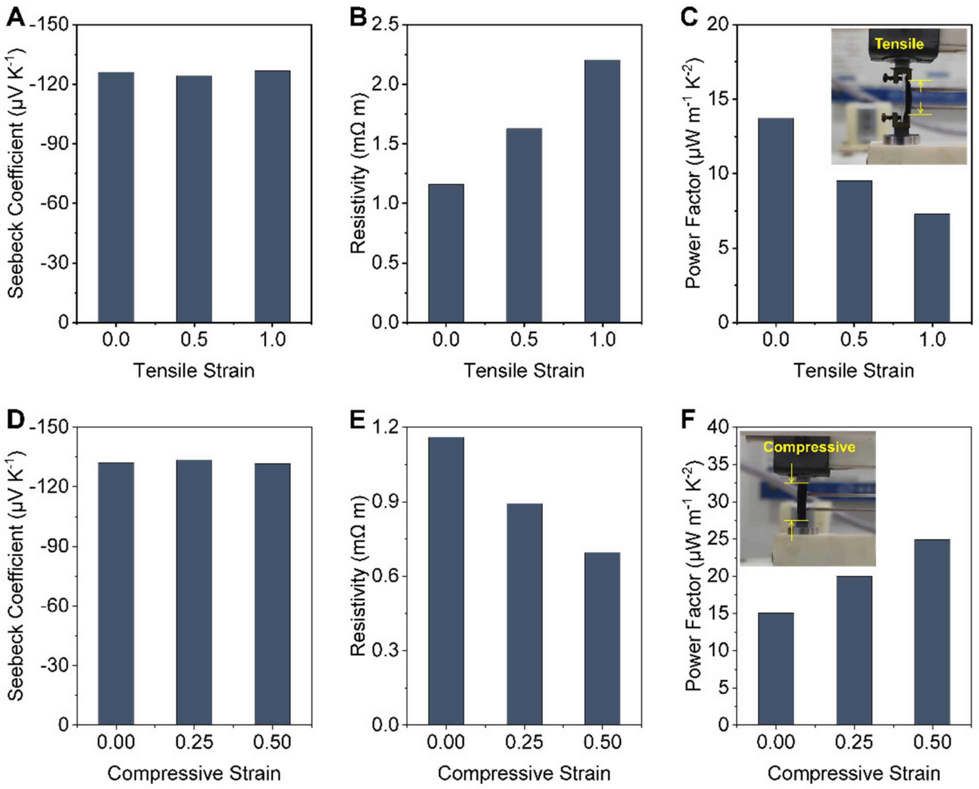

Fig. S4. The room-temperature Seebeck coefficient (**A, D**), resistivity (**B, E**) and power factor (**C, F**) of the Ag_2Se network under different strains.

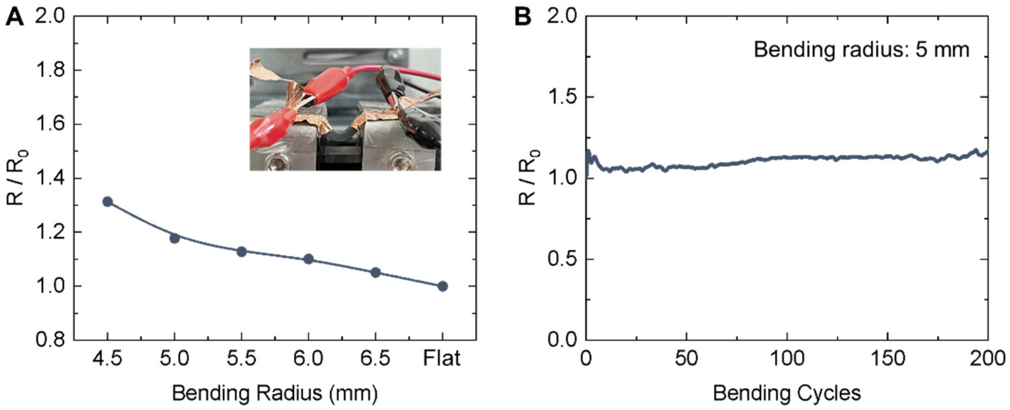

Fig. S5. **A**, Bending radius-dependent and **B**, bending cycles-dependent normalized resistance of the proposed Ag_2Se network.

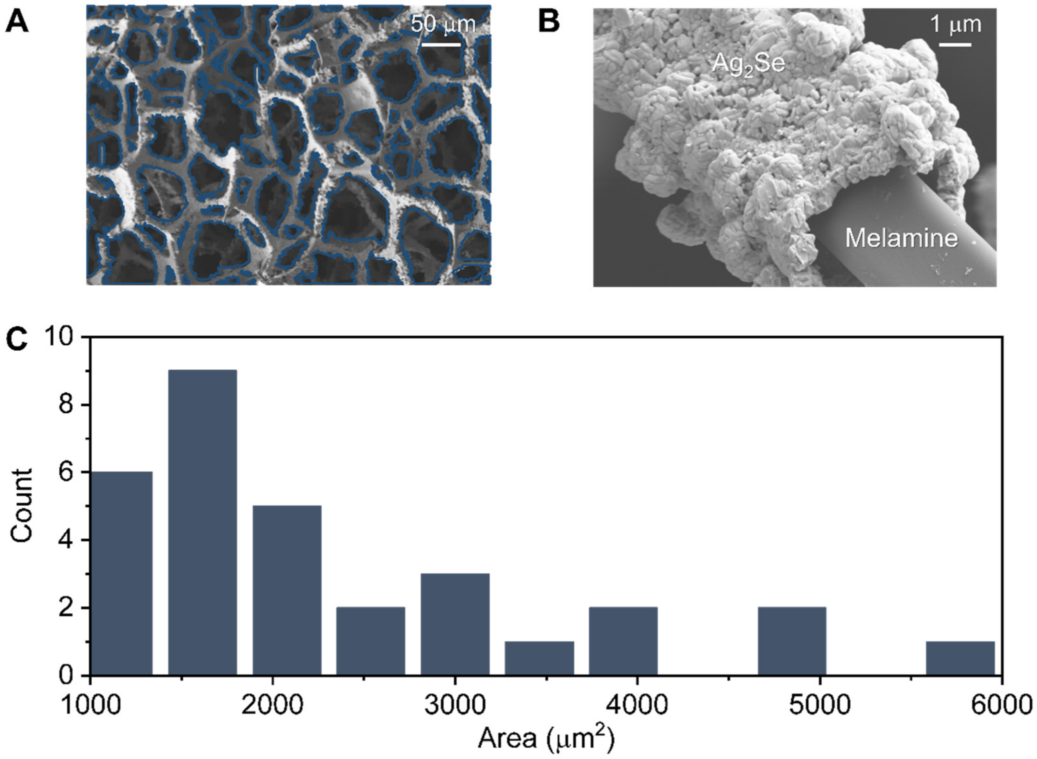

Fig. S6. **A**, The cross-section morphology of Ag_2Se network. **B**, A detailed image of Ag_2Se wrapping on a melamine fiber. **C**, Distribution of the pore size.

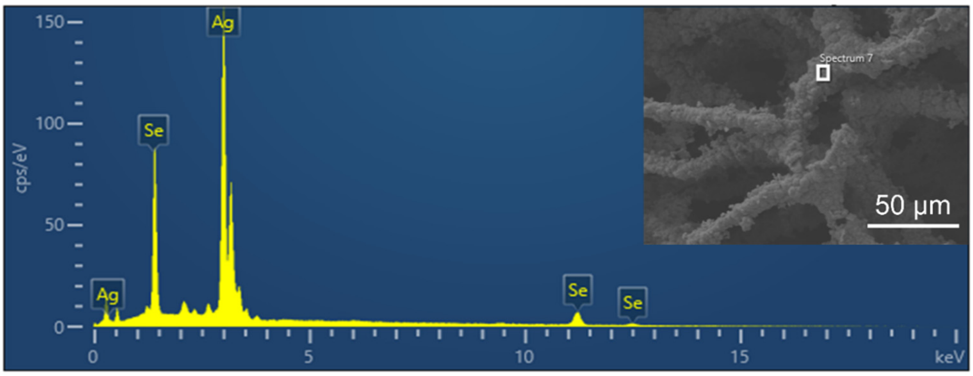

Fig. S7. Energy dispersive spectrum of Ag_2Se network**.**

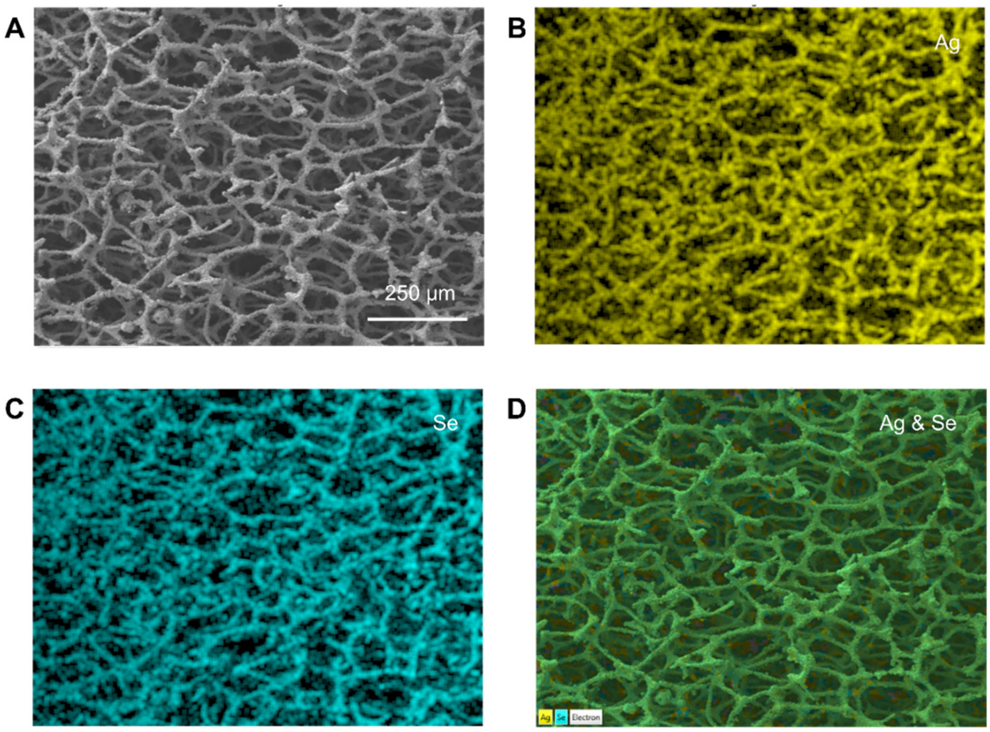

Fig. S8. The distribution of elements in Ag_2Se network.

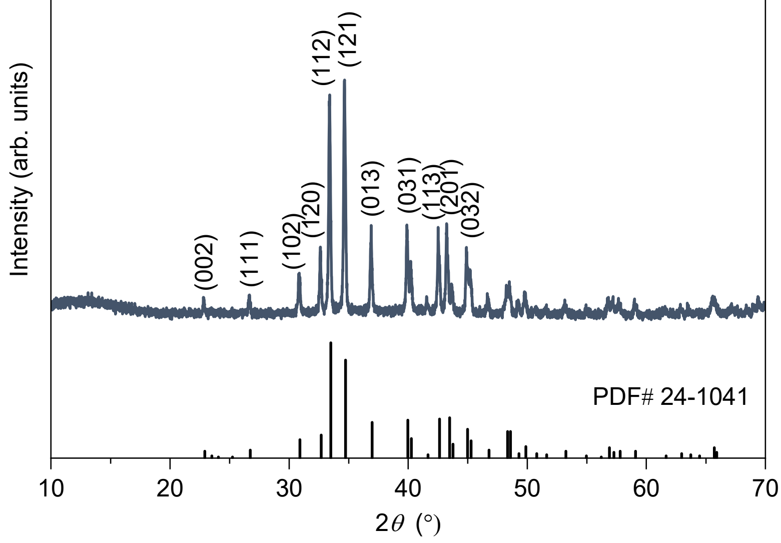

Fig. S9. XRD pattern of Ag_2Se network. The obtained Ag_2Se network is single phase without any impurities.

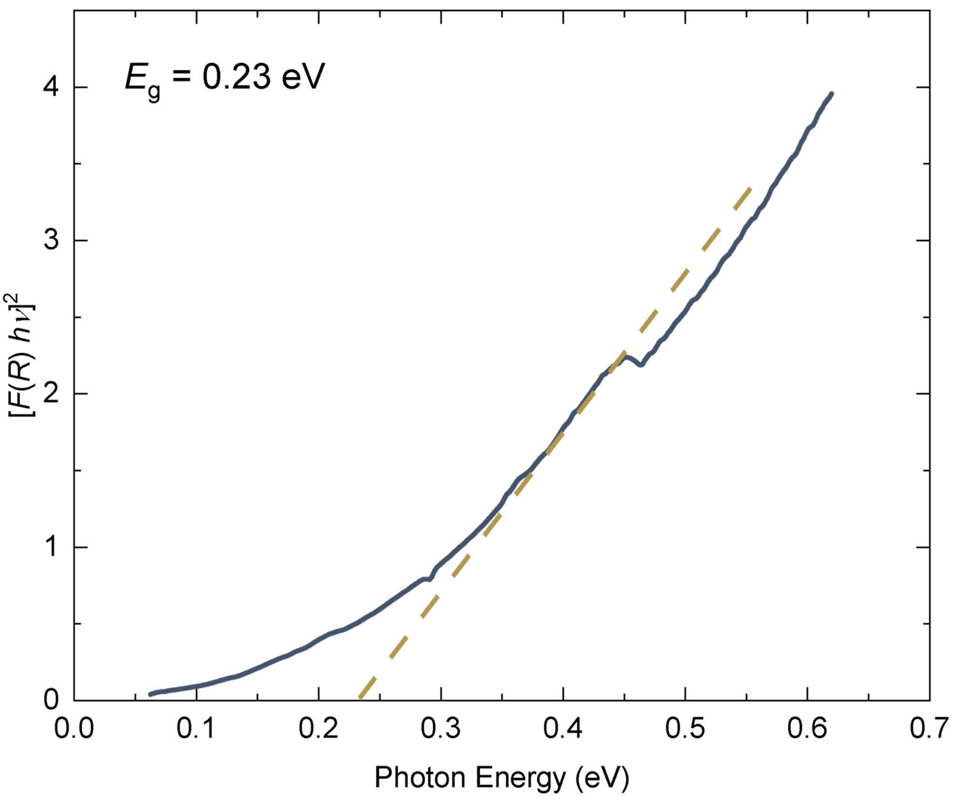

Fig. S10. Kubelka-Munk transformed reflectance spectra of Ag_2Se network.

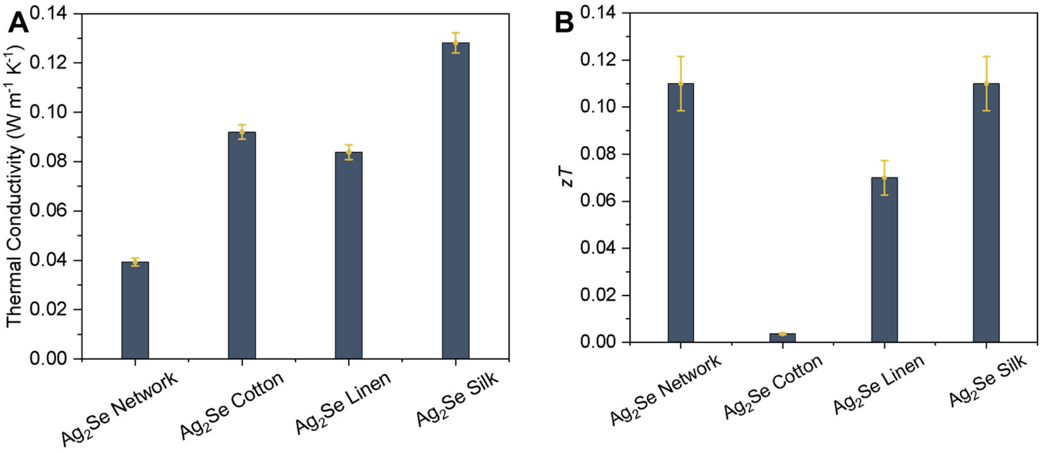

Fig. S11. **A**, Thermal conductivity and **B**, Room-temperature zT of the Ag_2Se network and Ag_2Se fabrics. Bars and error bars show the mean of the three readings and uncertainty of the results, respectively.

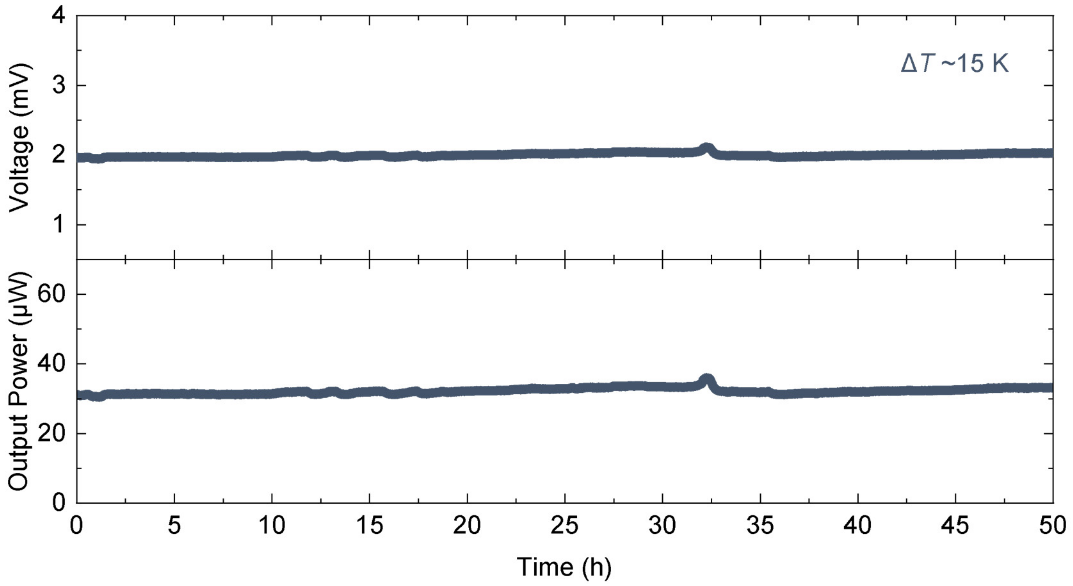

Fig. S12. The output performance changes with time at a fixed temperature difference of ~15 K.

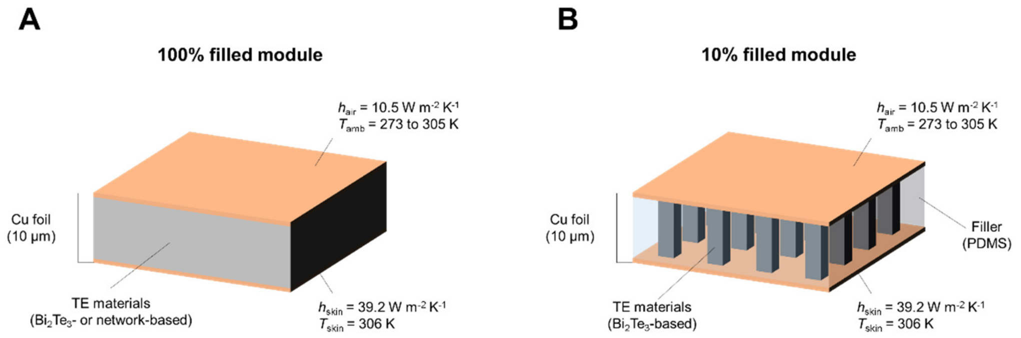

Fig. S13. Schematics of the simulation models. **A**, The Bi_2Te_3-based or Ag_2Se network based module with a fill factor of 100%. **B**, The Bi_2Te_3-based module with a fill factor of 10%. The specific material properties and the parameters' source were provided in Table S2.

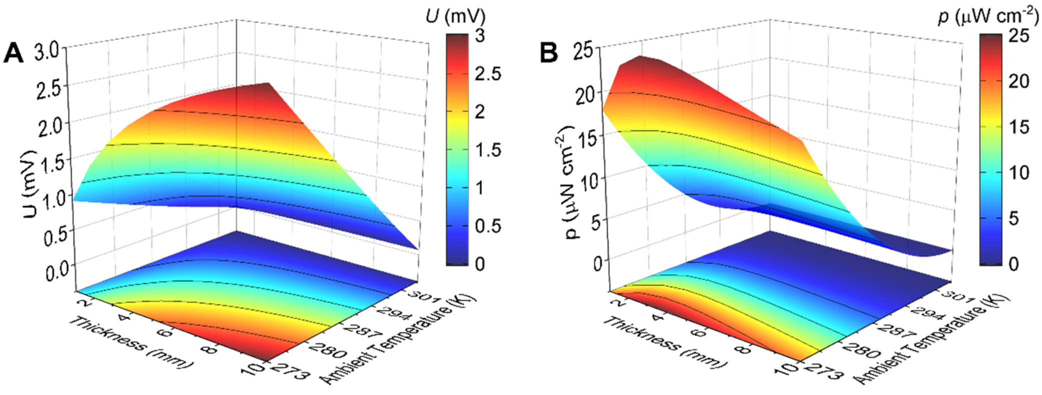

Fig. S14. **A**, Open-circuit voltage (*U*) and **B**, power density (*p*) of the network-based FTEG with different module thickness and ambient temperature.

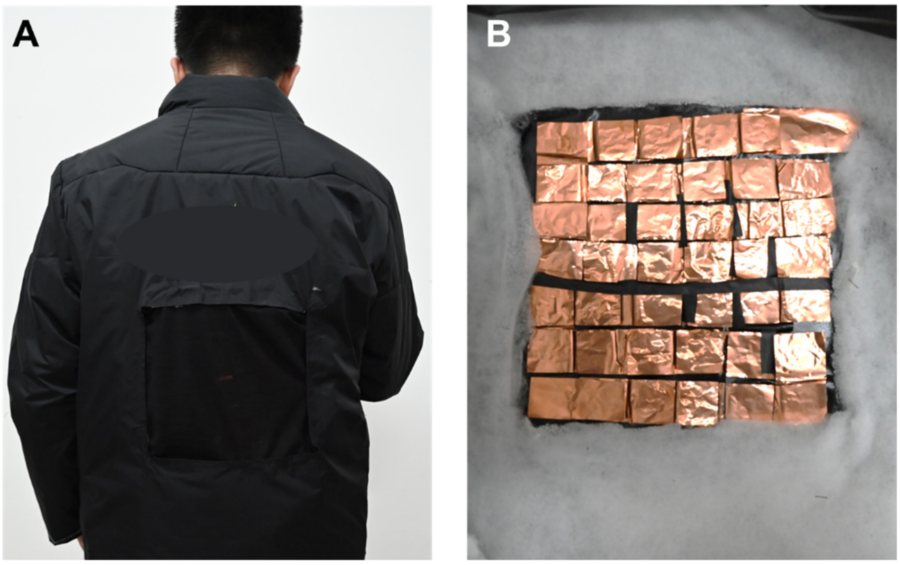

Fig. S15. **A**, Thermoelectric jacket. **B**, The padded network-based FTEG.

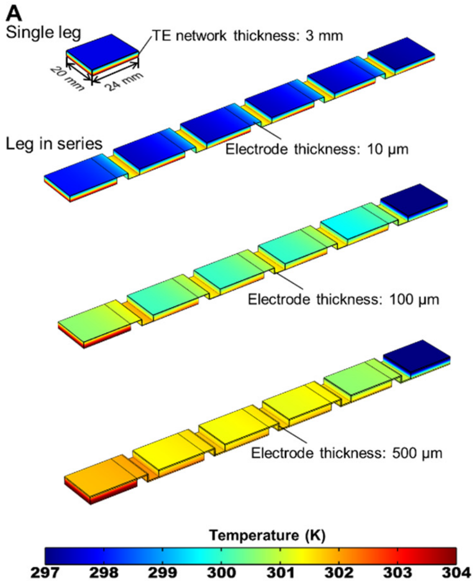

Fig. S16. Temperature distribution (**A**) and potential distribution (**B**) of the single leg and 6 legs in series. To further explain the effect of electrode thickness on temperature difference, we simulated the device with the same situation but thicker electrodes (*eg*., 100 μm and 500 μm). The device with thicker electrodes is difficult to establish temperature and potential differences.

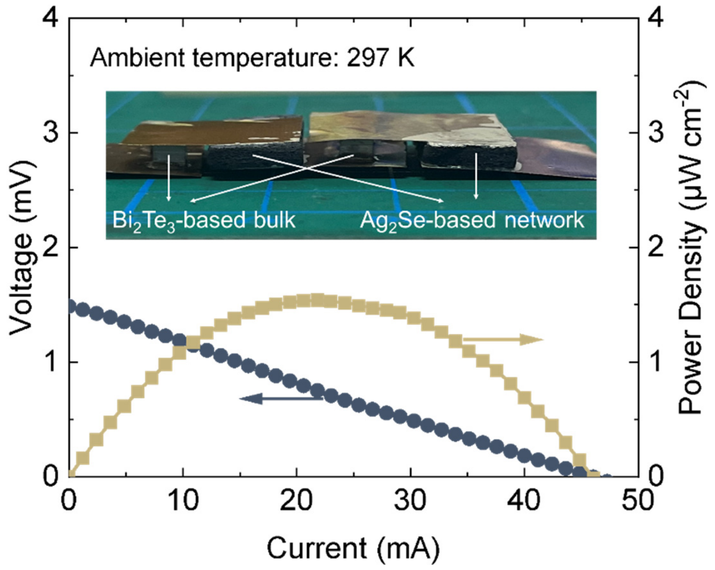

Fig. S17. Output performance of the FTEG with p-type Bi_2Te_3-based bulks and n-type Ag_2Se-based networks.

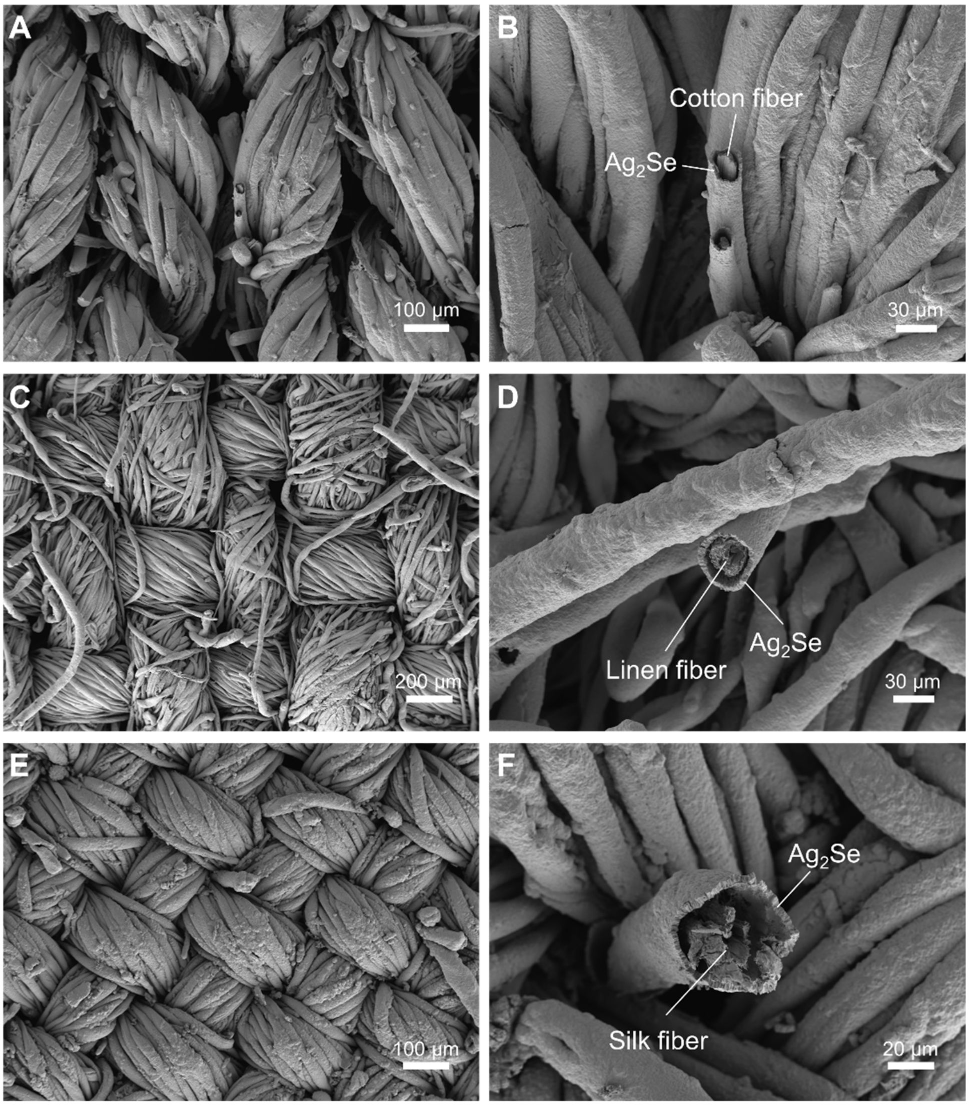

Fig. S18. The micrographs of various thermoelectric fabrics. **A** and **B**, Cotton fabrics. **C** and **D**, Linen fabrics. **E** and **F**, Silk fabrics.

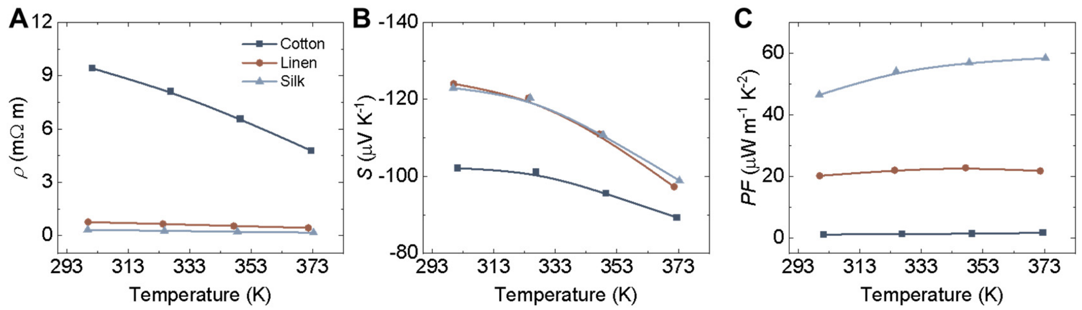

Fig. S19. Electrical resistivity (**A**), Seebeck coefficient (**B**) and power factor (**C**) of the thermoelectric textile.

Table S1. The element ratio in prepared Ag_2Se network.

Element	Wt%	Atomic%
Se	26.07	32.51
Ag	73.93	67.49
Total:	100.00	100.00

Table S2. The simulation conditions and parameters.

**Parameters**	**Values**	**Source**
Thermal conductivity of Ag_2Se network	0.04 W m^{-1} K^{-1}	Measurement
Thermal conductivity of TE bulk (commercial)	1.6 W m^{-1} K^{-1}	Ref.[1]
Thermal conductivity of copper	400 W m^{-1} K^{-1}	COMSOL
Thermal conductivity of filler (PDMS)	0.15 W m^{-1} K^{-1}	Ref.[2,3]
Resistivity of Ag_2Se network	1.16×10^{-3} Ω m	Measurement
Resistivity of TE bulk (commercial)	9.9×10^{-6} Ω m	Ref.[1]
Resistivity of copper	1.7×10^{-8} Ω m	COMSOL
Seebeck coefficient of Ag_2Se network	-130 μV K^{-1}	Measurement
Seebeck coefficient of TE bulk (commercial)	-197 μV K^{-1}	Ref.[1]
Heat transfer coefficient of skin surface	39.2 W m^{-2} K^{-1}	Ref.[3,4]
Heat transfer coefficient of air	10.5 W m^{-2} K^{-1}	Ref.[4]
Skin surface temperature	306 K	Ref.[3,4]
Module height (full filled / 10% filled / networked)	3 mm	–
Fill factor (full filled / 10% filled / networked)	100% / 10% / 100%	–

References

1 Liu, Y. *et al.* Passive Radiative Cooling Enables Improved Performance in Wearable Thermoelectric Generators. *Small* **18**, e2106875 (2022).

2 Suarez, F. *et al.* Flexible Thermoelectric Generator Using Bulk Legs and Liquid Metal Interconnects for Wearable Electronics. *Appl. Energy* **202**, 736-745 (2017).

3 Ozturk *et al.* Designing Thermoelectric Generators for Self-Powered Wearable Electronics. *Energy Environ. Sci.* (2016).

4 Kim, C. S. *et al.* Structural Design of a Flexible Thermoelectric Power Generator for Wearable Applications. *Appl. Energy* **214**, 131-138 (2018).
